# Spatial structure of yeast biofilms and the role of cell adhesion across different media

**DOI:** 10.1016/j.bioflm.2025.100306

**Published:** 2025-07-14

**Authors:** Vichi Sicha Irianto, Vítězslav Plocek, Rashim Bharti, Jana Maršíková, Libuše Váchová, Zdena Palková

**Affiliations:** a Faculty of Science, Charles University, BIOCEV, Prague, Czech Republic; b Institute of Microbiology of the Czech Academy of Sciences, BIOCEV, Prague, Czech Republic

**Keywords:** Yeast, Biofilm, Adhesion, Spatial structure, Microscopy

## Abstract

The ability of yeast cells to adhere to solid surfaces or even penetrate semi-solid surfaces and form multicellular biofilms are critical factors in infection. This study examines the relationship between cell adhesion capability and the ability to create spatially organized biofilms in selected *Saccharomyces cerevisiae* strains, including clinical isolates, and five *Candida* species (*C. albicans*, *C. glabrata*, *C. krusei*, *C. parapsilosis*, and *C. tropicalis*). We assessed cell adhesion to polystyrene surface in four media varying in source of carbon and other nutrients. Using microscopy of vertical cell arrangement profiles within yeast populations grown at the solid-liquid interface, we evaluated their internal organization to determine whether the populations exhibit typical biofilm characteristics, such as the spatial organization of distinct cell types. Results indicate that well adherent *S. cerevisiae* strains form spatial biofilms with typical internal organization, highlighting strain-specific responses to media composition and supporting the use of natural *S. cerevisiae* strains for biofilm research. Among *Candida* species, biofilm formation did not consistently align with adhesion efficiency to plastic; while *C. albicans* and *C. krusei* formed spatially structured biofilms on media where they adhered well, *C. tropicalis* and *C. glabrata* exhibited efficient adhesion without biofilm structuring. Interestingly, *C. parapsilosis* formed a structured biofilm despite minimal adhesion. These findings emphasize the role of media composition, particularly components of yeast extract and defined medium for mammalian cell growth RPMI, which differentially impacted adhesion and biofilm formation in *S. cerevisiae* and *C. albicans*.

## Highlights

• Vertical cross-sections show the internal organization of yeast biofilms over their entire thickness.
• Biofilm formation does not always correlate with adhesion strength, especially in *Candida* spp.
• Media components influence the complexity of biofilms differently in different yeast species.
• The yeast *S cerevisiae* can form a fully developed structured biofilm.

## 1 Introduction

The biofilm lifecycle, whether yeast or bacterial and developing on solid or semi-solid substrates, comprises four main stages [1,2]. It begins with cellular adhesion to a solid surface (such as plastic) or invasion into a semi-solid substrate (such as agar or tissue) (stage 1). This is followed by stages 2–3 during which cells attached to the substrate establish a basis for the formation of a multilayered three-dimensional structure. This structure is typically composed of various differentiated cells that are spatially organized, such as yeast-form oval cells, hyphae, or pseudohyphae. A mature biofilm includes also a non-cellular extracellular matrix (ECM), which is essential for the biofilm structural integrity and resistance to external factors. In addition to structural support, the ECM—together with surface adhesins—mediates cell–cell interactions within the biofilm, thus contributing to the architecture of the 3D structure. The final stage (stage 4) is the partial dispersal of the biofilm, releasing planktonic cells.

*Candida albicans* is the most extensively studied yeast with respect to its biofilm-forming ability [1,3,4]. It is also the prevalent opportunistic yeast pathogen and its ability to form a biofilm is an important risk factor for yeast-associated infections [[5], [6], [7]]. In addition to *C. albicans*, opportunistic yeast pathogens also include *C. glabrata*, *C. parapsilosis*, and *C. tropicalis*, as well as some less-studied yeasts, such as *C. krusei* [8]. Several of these species, such as *C. tropicalis*, *C. parapsilosis* and *C. krusei*, have also been shown to form biofilms [9,10]. However, the ability to form biofilms in other yeast species such as *S. cerevisiae* and *C. glabrata* has remained uncertain or questioned, and is mainly based on reports of adhesion of these species to abiotic surfaces such as polystyrene, glass or cotton fibers [[10], [11], [12], [13], [14]]. The ability to form complex three-dimensional biofilms on polystyrene surfaces was recently documented in a natural *S. cerevisiae* strain [15]. Since biofilm formation can be influenced by growth conditions, the inability to form spatial biofilms attributed to some yeasts could be due to different conditions used to test biofilm formation in different studies [5]. Furthermore, as observed in *S. cerevisiae*, only certain strains form biofilms [15,16].

There is also limited information regarding differences in the structural organization of biofilms formed by different yeasts. Biofilm formation is often detected using methods that measure biomass accumulation after removal of non-adherent cells [17] or detect the activity of adherent biofilm cells with tetrazolium salt [18], methods that do not provide structural data on the 3D composition of the biofilm.

Since sugar concentrations (such as glucose and other hexoses, or alternative carbon sources) vary significantly in different niches that yeast can colonize (including the human body), the regulatory pathways and networks governing carbon metabolism must be able to respond to the local availability of these nutrients and adapt to changes [19,20]. Carbohydrate metabolism, as well as the metabolism of other nutrients, is also closely linked to the ability of yeasts to switch between different cell types, such as yeast-shaped cells and hyphae or pseudohyphae, particularly in *C. albicans* [19]. The metabolism of various carbon sources (different sugars) and their effect on the behavior and phenotype of *S. cerevisiae* is relatively well understood [15,[21], [22], [23]]. In *Candida* species, changes in carbon metabolism can significantly influence yeast virulence attributes, including cell adhesion, morphogenesis, invasion, oxidative stress resistance, biofilm formation and antifungal drug tolerance [19,20]. The most extensive information on sugar metabolism, its regulation, and its impact on behavior relates primarily to *C. albicans* and, to a lesser extent, to *C. glabrata*. Some regulatory mechanisms are similar to those in *S. cerevisiae*, while others differ. For example, unlike *S. cerevisiae*, which shifts to fermentative growth in the presence of high glucose, *C. albicans* is capable of assimilating glucose and other carbon sources simultaneously [24]. Information on carbon metabolism in other *Candida* species is largely limited.

In this study, we aimed to determine whether the ability of yeast cells to efficiently adhere to plastic surfaces is a sufficient prerequisite for the formation of a spatially structured and differentiated biofilm. Following the biofilm life cycle, we define here a mature biofilm as a three-dimensional structure composed of multiple cell types occupying distinct spatial regions. We also investigated whether the capacity for cell-to-surface adhesion and biofilm formation under different growth conditions is a general trait of a given yeast species or whether it is strain-specific within the same species.

## 2 Results

### 2.1 Cell adhesion to polystyrene surface varies across different *S. cerevisiae* strains and is affected by medium composition

**Fig. 1:**
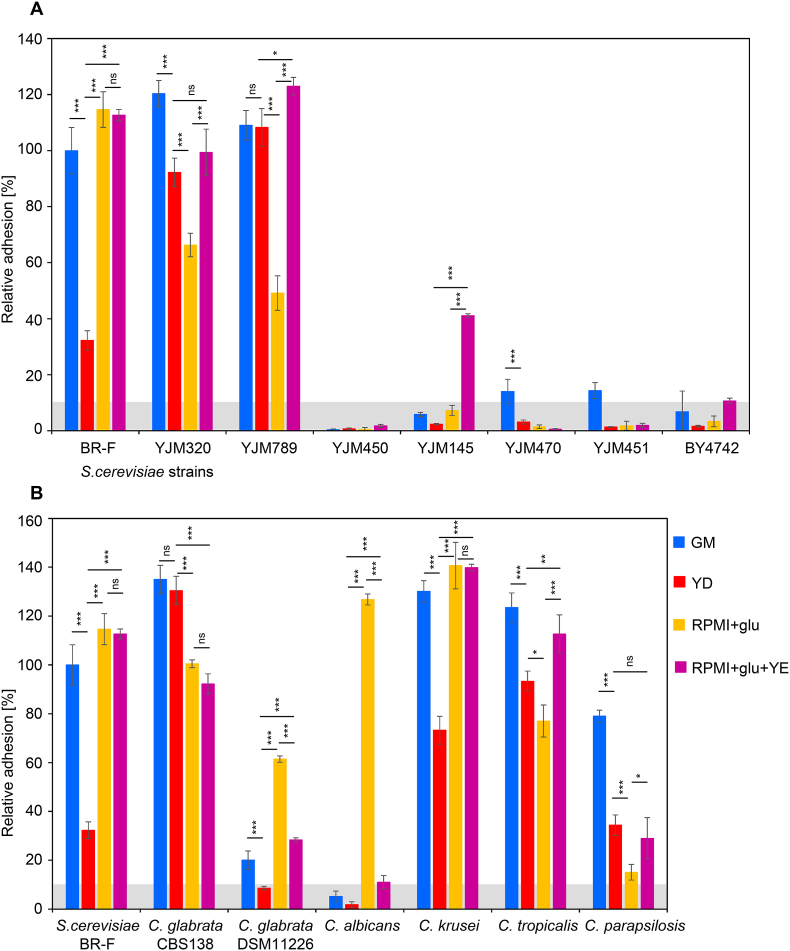
*Saccharomyces* strain and *Candida* spp. cell adhesion on plastic in different media. Relative values of adhesion of different *S. cerevisiae* strains (A) and different *Candida* spp. (B) to plastic relative to adhesion of strain BR-F grown in respiratory GM medium that was set to 100 %. Average of 4 independent cell replicates is shown, with standard deviation (SD) calculated. The significance of differences was determined using a one-way ANOVA with Tukey post test for multiple comparisons: ∗∗∗, p < 0.001; ∗∗, p < 0.01 and ∗, p < 0.05. The values in the gray zone (<10 % adherence of the BR-F strain to GM) are low and may be inaccurate, we therefore consider them indicative and were not statistically evaluated.

Previously it was shown that the wild strain *S. cerevisiae* BRAIN 97-fluffy (BR-F) [25] (Table 1) adheres to solid plastic surfaces and forms a biofilm, and that this adhesion is significantly repressed in the presence of glucose [15]. In this study, we first focused on the analysis of cell adhesion to plastic of six clinical isolates of *S. cerevisiae*, compared to the adherent BR-F strain and a non-adherent laboratory strain, BY4742 (Fig. 1A) (Table 1). Adhesion was quantified using a modified crystal violet staining assay followed by colorimetric measurement of the dye (see Sections 4.2, 4.3). Absorbance at 592 nm was measured and, throughout this study, values were expressed as percentages relative to the values measured for BR-F strain grown in the GM respiratory medium, which was set to 100 %.

**Table 1:** List of the strains used in the study.

Yeast	Strain	Source	Collection No.
*S. cerevisiae*	BY4742	Euroscarf	Y10000
*S. cerevisiae*	BR-F	Collection of the Institute of Chemistry, Slovak Academy of Sciences	CCY 21-4-97
*S. cerevisiae*	YJM320	Phaff Yeast Culture Collection	11–133
*S. cerevisiae*	YJM789	Phaff Yeast Culture Collection	15–436
*S. cerevisiae*	YJM450	Phaff Yeast Culture Collection	15–410
*S.cerevisiae*	YJM145	Phaff Yeast Culture Collection	09–109
*S. cerevisiae*	YJM470	Phaff Yeast Culture Collection	11–141
*S. cerevisiae*	YJM451	Phaff Yeast Culture Collection	15–311
*C. glabrata*	CBS138	ATCC collection	2001
*C. albicans*	DSM11225	DSMZ collection	11,225
*C. glabrata*	DSM11226	DSMZ collection	11,226
*C. krusei*	DSM6128	DSMZ collection	6128
*C. tropicalis*	DSM5991	DSMZ collection	5991
*C. parapsilosis*	DSM5784	DSMZ collection	5784

Adhesion assays were conducted with cells pre-grown in four media types that differ in complexity and carbon source. Two of these media, respiratory medium GM (a complex medium containing glycerol and yeast extract [YE]) and glucose medium YD (a complex medium containing 2 % glucose and YE) differed only in the carbon source. These media were selected based on previously observed differences in adhesion of the BR-F strain [15] (see also Fig. 1A). The third medium, RPMI 1640 with glucose (RPMI+glu), is a synthetic defined medium, which has been commonly used for analysis of cell adhesion and biofilm formation in different *Candida* spp. The glucose supplement in RPMI differed between studies, being either 0.2 %, 0.4 % or 2 % glucose [[26], [27], [28]]. In our study, we used 2 % glucose, the same concentration as in YD medium. The fourth medium (RPMI+glu+YE) was RPMI+glu further supplemented with 1 % YE, matching the YE concentration in YD. Thus, RPMI+glu+YE differed from YD medium only in the presence of RPMI components. For all strains, we optimized pre-cultivation growth conditions to prevent glucose depletion in the YD or RPMI+glu medium prior to the adhesion experiments. The goal was to ensure that cells were still in the growth phase using glucose as their primary carbon source and had not yet undergone the diauxic shift - a metabolic transition well described in *S. cerevisiae*, in which glucose is exhausted and cells switch to utilizing secondary carbon sources such as ethanol. This metabolic shift is typically associated with substantial metabolic reprogramming [[29], [30], [31], [32]], which may also impact cellular adhesion properties.

As expected, strain BR-F grown in GM medium showed strong adhesion, whereas adhesion was markedly reduced in cells grown in glucose-containing YD medium (Fig. 1A). Surprisingly, however, BR-F adhered comparably well in RPMI+glu (2 % glucose, identical to the concentration in YD) as in GM. Among the six clinical isolates of *S. cerevisiae*, two strains (YJM320 and YJM789) adhered similarly to the BR-F strain in respiratory medium GM, but unlike BR-F, they also showed significant adhesion in glucose medium YD. However, adhesion was significantly reduced in RPMI+glu, especially in strain YJM789. The other four clinical strains of *S. cerevisiae* exhibited minimal adhesion across all three media tested. As expected, the laboratory strain BY4742 did not adhere to plastic in any of the media.

Given the observed differences in adhesion for strain BR-F and the two clinical isolates in the two glucose-containing media (YD and RPMI+glu) (Fig. 1A), we conducted further analyses using a modified medium. Specifically, we supplemented RPMI+glu with YE to a 1 % concentration, matching that in YD medium. Due to the faster growth rate of all strains in this combined RPMI+glu+YE medium, we adjusted the pre-cultivation period before adhesion experiments to ensure glucose levels remained comparable to those in previous experiments where strains were cultivated in YD or RPMI+glu media.

Strain BR-F adhered comparably in RPMI+glu+YE medium to its adhesion in GM and RPMI+glu, showing significantly higher adhesion than in YD medium. Both clinical strains YJM320 and YJM789, which were adhesive to plastic when grown in all three previous media types, also showed efficient adhesion in RPMI+glu+YE. For both, adhesion in RPMI+glu+YE was significantly higher than in RPMI+glu alone. The difference between RPMI+glu and RPMI+glu+YE was more pronounced in strain YJM789. Surprisingly, one of the generally non-adhesive clinical strains, strain YJM145, reproducibly exhibited moderate adhesion only in combined RPMI+glu+YE medium (Fig. 1A).

In summary, different adhesive *S. cerevisiae* strains BR-F, YJM320 and YJM789 reacted differently to medium composition, and exhibited different adhesion profiles. All these strains were consistently adhesive in respiratory GM and combined RPMI+glu+YE media. However, the RPMI components enhanced adhesion of the BR-F strain grown with glucose, while they reduced the adhesion of the two clinical isolates (YJM320, YJM789) in RPMI+glu. The addition of yeast extract in RPMI+glu counteracts this negative effect for both clinical isolates. In contrast, the three clinical strains (YJM450, YJM470 and YJM451) showed no detectable adhesion under any of the conditions tested (Fig. 1A). These results underscore the considerable variability in adhesion ability among natural and clinical *S. cerevisiae* strains, even when cultivated under identical environmental conditions. This strain-level variability highlights the importance of evaluating phenotypes at the level of individual isolates. Generalizing cellular behavior at the species level may obscure functionally and biologically relevant differences.

### 2.2 Prominent differences in Candida spp. adhesion depending on medium composition

We next focused on comparing the cell adhesion properties of five *Candida* species (Table 1), which are opportunistic yeast pathogens. We included two strains of *C. glabrata* (CBS138, isolated from feces, and DSM11226, isolated from blood), and strains of *C. albicans* (DSM11225, isolated from human blood), *C. krusei* (DSM6128, isolated from a sputum sample of a patient with bronchomycosis), *C. parapsilosis* (DSM5784, isolated from a human case of sprue), and *C. tropicalis* (DSM5991). As with *S. cerevisiae*, we examined the relative adhesion to plastic of these *Candida* strains across four media - GM, YD, RPMI+glu, and RPMI+glu+YE - using BR-F grown in GM medium as the reference (set to 100 %) (Fig. 1B).

The most striking medium-dependent effect was observed in the *C. albicans* strain, which showed no adhesion in either the respiratory GM or glucose-based complex YD media, but displayed high adhesivity in RPMI+glu. The addition of yeast extract to RPMI+glu completely inhibited *C. albicans* adhesion.

*C. glabrata* CBS138 adhered in all four media (Fig. 1B), whereas *C. glabrata* DSM11226 exhibited minimal adhesion in GM, YD and RPMI+glu+YE, with greater adhesion in RPMI+glu, a pattern somewhat similar to that of *C. albicans*. *C. krusei* showed high adhesion in GM and RPMI+glu and exhibited slightly lower, though still significant, adhesion in YD (values ∼2 times higher than that of BR-F). This adhesion pattern resembles that of the *S. cerevisiae* BR-F strain. Similar to BR-F, the addition of YE to RPMI+glu did not reduce *C. krusei* adhesion; adhesion levels in RPMI+glu+YE remained comparable to those in RPMI+glu and were significantly higher than in YD.

*C. tropicalis* exhibited the highest adhesion in respiratory medium GM, with slightly reduced adhesion in YD and RPMI+glu. However, adding YE to RPMI+glu increased *C. tropicalis* adhesion to levels comparable with GM, and thus higher than in YD and RPMI+glu. A similar profile was observed for *C. parapsilosis* in GM, YD, and RPMI+glu, though it showed overall lower adhesion, with lowest RPMI+glu adhesion of all *Candida* strains analyzed. When YE was added, *C. parapsilosis* adhesion increased but only to levels similar to those in YD and slightly higher than in RPMI+glu.

These findings suggest that YE strongly inhibits the adhesion of *C. albicans* (and partially *C. glabrata* DSM11226), both of which adhered only when pre-grown in RPMI+glu without YE. In contrast, similar to the pattern seen in *S. cerevisiae* BR-F, the presence of RPMI components appears essential for enhancing *C. krusei* adhesion in the presence of glucose, as evidenced by the doubling of adhesion in RPMI+glu+YE compared to YD - in media differing only by the presence of RPMI components.

### 2.3 Colony/biofilm structures on YD, GM and RPMI+glu agar compared to structures in microtiter wells

**Fig. 2:**
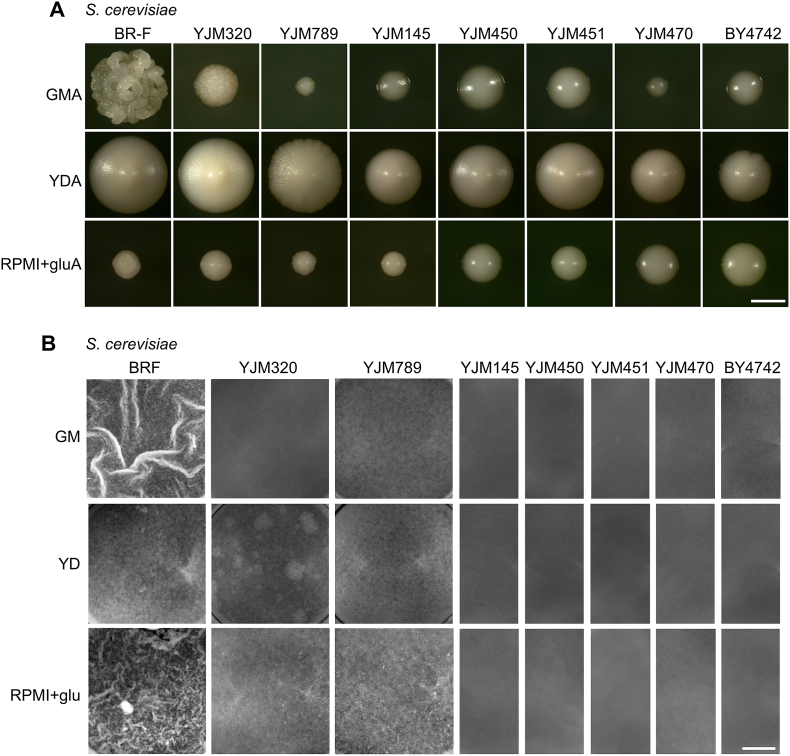
Morphology of colonies, colony biofilms and SLI biofilms formed by *S. cerevisiae* strains. (A) *S. cerevisiae* strains were grown on GMA, YDA or RPMI+gluA plates for 3 days. Each structure, smooth colony or wrinkled colony biofilm is shown at the same magnification. (B) *S. cerevisiae* strains were grown in GM, YD or RPMI+glu medium in microtiter wells for 24 h. Images of the microtiter wells show the morphology of the structure from the top. Wrinkles are visible in BR-F (GM and RPMI+glu) SLI biofilms. Bar, 1 mm.

**Fig. 3:**
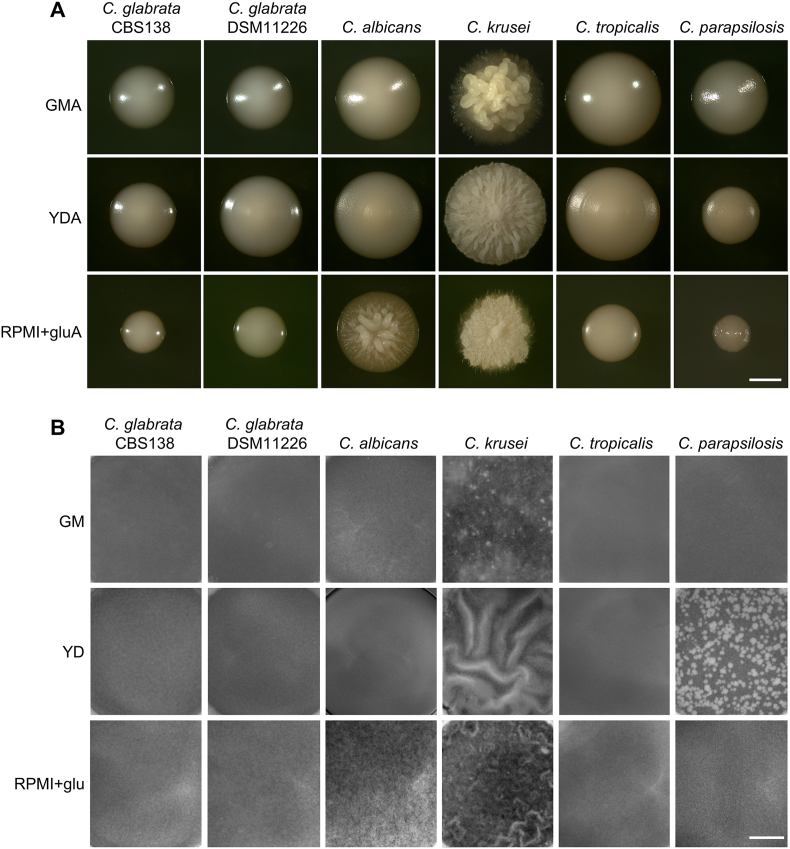
Morphology of colonies, colony biofilms and SLI biofilms formed by *Candida* spp. strains. (A) *Candida* spp. strains were grown on GMA, YDA or RPMI+gluA plates for 3 days. Each structure, smooth colony or wrinkled colony biofilm is shown at the same magnification. (B) *Candida* spp. strains were grown in GM, YD or RPMI+glu medium in microtiter wells for 24 h. Images of the microtiter wells show the morphology of the structure from the top. Wrinkles are visible in *C. krusei* (YD and RPMI+glu) SLI biofilms. Bar, 1 mm.

*S. cerevisiae* BR-F strain forms wrinkled colony biofilms on respiratory GM agar (GMA), while the laboratory strain BY4742 forms smooth colonies on the same medium [[33], [34], [35]]. Both strains form smooth colonies on YD agar (YDA) (Fig. 2A). To preliminarily assess the ability of the strains to form spatially wrinkled populations, we observed the three-day colony morphology of all tested strains (*S. cerevisiae* and *Candida* spp.) on GMA, YDA and RPMI+glu agar (Fig. 2, Fig. 3A). In parallel, we examined the structures formed within GM, YD and RPMI+glu liquid media in microtiter wells after 24 h of cultivation (Fig. 2, Fig. 3B), a time sufficient for the BR-F strain to form wrinkled solid-liquid interface (SLI) biofilms in GM medium [15].

In addition to *S. cerevisiae* BR-F strain, only *C. krusei* and, to a lesser extent, the clinical *S. cerevisiae* strain YJM320, formed wrinkled structures on GMA (with YJM320 exhibiting less pronounced external structuring) (Fig. 2, Fig. 3A). Unlike the other yeast strains, *C. krusei* also formed distinctively wrinkled colonies on YDA. On RPMI+glu agar, *C. albicans* and BR-F formed wrinkled colonies. *C. krusei* colonies on RPMI+glu agar were not typically wrinkled but exhibited a distinctive “hairy” surface texture (Fig. 2, Fig. 3A). All remaining strains formed smooth colonies lacking visible macroscopic structuring. Most *S. cerevisiae* strains (including BR-F) grew slowly on this agar.

In microtiter wells, BR-F formed wrinkled SLI structures in both GM and RPMI+glu media, and wrinkles were also observed in *C. krusei* in YD and RPMI+glu media (Fig. 2, Fig. 3B). Among other *S. cerevisiae* strains, some surface heterogeneity was visible in BR-F on YD medium, and in the adhesive clinical strains YJM320 (in YD and RPMI+glu) and YJM789 (in all media). The surfaces of all other non-adhesive *S. cerevisiae* cell populations appeared visually homogeneous in all media (Fig. 2B).

In the *Candida* species, surface homogeneity or heterogeneity of the SLI biofilm on plastic did not consistently correlate with strain adhesiveness. For example, the adhesive strain *C. tropicalis* formed a visually homogeneous surface in all tested media, and only slight heterogeneity was observed in the adhesive *C. glabrata* CBS138 strain (in YD and RPMI+glu). Most prominent heterogeneity was observed in highly adhesive strains *C. krusei* (in all media) and *C. albicans* (in RPMI+glu), but also in the low-adherent *C. parapsilosis* (in YD) (Fig. 3B).

These results suggest that macroscopically observable wrinkling (*S. cerevisiae* and *C. albicans*) or other surface heterogeneity (such as the hairy structures of *C. krusei*) in agar-grown populations typically correlates with the presence of wrinkling or heterogeneity in SLI biofilms (Fig. 2, Fig. 3). An exception was *C. parapsilosis*, which formed smooth colonies on YDA but exhibited surface heterogeneity in SLI populations grown in YD (Fig. 3B). On the other hand, these structural features did not strictly correlate with the efficiency of cell adhesion, particularly in *Candida* species. For example, the low-adherent *C. parapsilosis* produced a heterogeneous SLI structure in YD, whereas the highly adherent *C. tropicalis* always formed smooth, homogeneous populations. However, the absence of macroscopic wrinkling or surface heterogeneity does not exclude the possibility of forming a spatially organized biofilm composed of distinct cell types.

### 2.4 Formation of spatially structured biofilms comprising different cell types does not necessarily correlate with efficient cell adhesion to plastic

Adhesion of yeast cells to a surface is a critical first step in the biofilm life cycle on solid and semi-solid surfaces [1,36]. This process is influenced by multiple factors, including non-specific physicochemical interactions such as van der Waals forces and hydrophobic interactions, as well as specific interactions mediated by cell wall-associated adhesion molecules [37]. Well-characterized examples include the agglutinin-like sequence (Als) protein family in *C. albicans* and the Flo11 adhesin in *S. cerevisiae* [[37], [38], [39]]. On semi-solid substrates, cell adhesion can be accompanied by cell penetration into the substrate (e.g., agar or tissue), which is often associated with dimorphic differentiation, hyphal or pseudohyphal formation, or at least formation of chains of unipolar budding yeast cells [[40], [41], [42], [43], [44]]. In the later stages of biofilm development, a spatial structure can form, comprising differentiated cells that can occupy specific spatial positions, as observed in colony biofilms of *S. cerevisiae* and certain *C. albicans* biofilms [10,[44], [45], [46], [47]]. At this stage, the ECM, along with intercellular interactions, plays an important role in the formation of the spatial structure of the biofilm. Some adhesins such as Flo11 and Als1, which mediate initial surface attachment, are also key factors in mediating cell-cell interactions in the spatial biofilm [37,38,44,48,49].

Given this background, we next examined whether the ability of yeast cells to adhere to plastic surfaces (Fig. 1) correlates with their ability to form spatially structured biofilms. We classified yeast populations exhibiting either a wrinkled spatial arrangement, as seen in *S. cerevisiae* BR-F biofilms (both colony and SLI biofilms), and/or internal cell heterogeneity characterized by distinct spatial localization of different cell types (e.g., yeast-form cells and hyphae/pseudohyphae), as spatially organized biofilm structures. In contrast, we classified compact, planar multilayer cell populations lacking visible internal cellular heterogeneity as unstructured cell layers (UCLs). These UCLs may consist of i) non-adherent cells that sediment and grow at the bottom of the microtiter well (e.g., *S. cerevisiae* laboratory strain BY4742), ii) a basal layer of adherent cells overlaid by growing non-adherent cells, or iii) a base of adherent and/or non-adherent cells with additional growth of mutually adherent cells. Due to morphological similarity among these three possible types of UCLs, our current approach cannot reliably distinguish between them. Importantly, none of these UCL types meet our criteria for spatially structured biofilms as defined above.

**Fig. 4:**
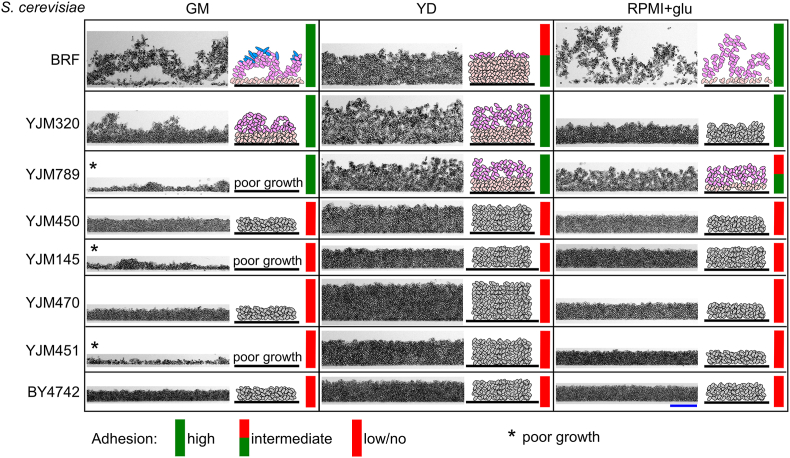
Spatially structured SLI biofilms and UCLs formed by different *S. cerevisiae* strains. *S. cerevisiae* strains were grown in microtiter plates at solid-liquid interface in GM, YD or RPMI+glu media for 24 h. Shown are representative parts of vertical cross-sections of biofilms or unstructured cell layers (UCLs) visualized by bright-field microscopy. The cell organization scheme is displayed on the right side of each microscopy image. It indicates whether the 3D population is structured and whether different cell types (indicated by different colors in the schemes) are present. Schematic representations in gray color indicate structures in which we have not observed morphologically different cell types (i.e. cell layers are formed by morphologically homogeneous cells). In schematic representations of differentiated structures/biofilms: oval cells in light pink, the basal biofilm layer; oval cells in differentiated upper biofilm layers in dark pink; elongated cells in blue. Black line, the plastic surface of the bottom of the well. The colored (red or green or red+green) bar in the right part of each population indicates whether the strain was adherent or not in the respective medium (based on the data from Fig. 1A). Blue bar, 100 μm. In each strain/condition, shown is a representative cross-section of a biofilm from the analysis of 4 biofilms, which were examined in 2 biological experiments (2 biofilms each). (For interpretation of the references to color in this figure legend, the reader is referred to the Web version of this article.)

**Fig. 5:**
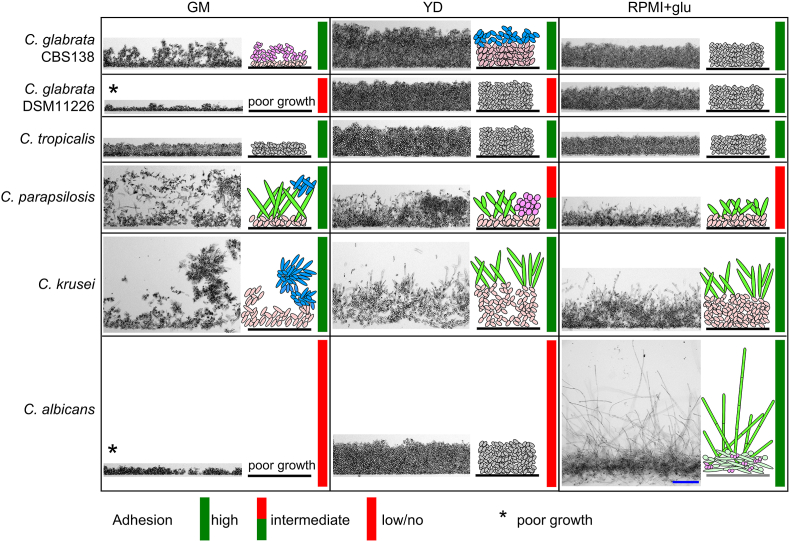
Spatially structured SLI biofilms and UCLs formed by different *Candida* spp. strains. *Candida* spp. strains were grown in microtiter plates at solid-liquid interface in GM, YD or RPMI+glu media for 24 h. Shown are representative parts of vertical cross-sections of biofilms or unstructured cell layers (UCLs) visualized by bright-field microscopy. The cell organization scheme is displayed on the right side of each microscopy image. It indicates whether the 3D population is structured and whether different cell types (indicated by different colors in the schemes) are present. Schematic representations in gray color indicate structures in which we have not observed morphologically different cell types (i.e. cell layers are formed by morphologically homogeneous cells). In schematic representations of differentiated structures/biofilms: Oval cells in light pink, the basal or lower layers; oval (or rounded) cells in differentiated upper biofilm layers in dark pink; elongated cells in blue; long hyphae or pseudohyphae in green. Black line, the plastic surface of the bottom of the well. The colored (red or green or red+green) bar in the right part of each population indicates whether the strain was adherent or not in respective media (based on the data from Fig. 1B). Blue bar, 100 μm. In each strain/condition, shown is a representative cross-section of a biofilm from the analysis of 4 biofilms, which were examined in 2 biological experiments (2 biofilms each). (For interpretation of the references to color in this figure legend, the reader is referred to the Web version of this article.)

In the experiments below, we analyzed the vertical organization of 24-h-old populations formed by individual yeast strains growing in microtiter plates in three different media (GM, YD, and RPMI+glu) (Fig. 4, Fig. 5). Vertical cross-sections of native populations were prepared using a method developed for *S. cerevisiae* biofilms [15], and examined microscopically using transmitted light microscopy. The laboratory strain BY4742—which lacks both surface and intercellular adhesion [15,49]—was used as a phenotypic control of UCLs. The adhesion or non-adhesion characteristics mentioned throughout chapters 2.4.1 and 2.4.2 refer to cell adhesion to plastic of each strain pre-cultured in the respective media, as shown in Fig. 1. This corresponds to the first cell-to-surface adhesion stage in the biofilm life cycle.

#### 2.4.1 Spatial structures of populations formed by different *S. cerevisiae* strains

Of the three *S. cerevisiae* strains that adhered to plastic in GM medium (Fig. 1), only the BR-F strain formed wrinkled biofilm structures (Fig. 2B), seen also on the vertical cross-sections (Fig. 4). The different morphologies of structures observed in the two adherent clinical strains may be partly due to suboptimal growth in GM respiratory medium (Fig. 4), which is particularly evident in strain YJM789, and is also visible in colony growth on GMA (Fig. 2A). The YJM320 strain grew more robustly than YJM789, but only faint structures slightly similar to wrinkles appeared in the surface cell layers. In YD medium, both adherent clinical strains formed a similar spatial structure, consisting of clusters of interconnected cells with intercellular spaces on the top of more densely arranged underlying cell layers. BR-F, which shows minimal adhesion to plastic in YD (Fig. 1), formed a more compact, homogeneous cell layers with minimal intercellular space (Fig. 4).

In RPMI+glu (Fig. 4), the highly adherent BR-F strain produced wrinkled structures similar to those in GM, though with fewer elongated cells. The moderately adherent clinical strain YJM789 produced structures in RPMI+glu similar to those observed in YD, while YJM320 formed a significantly more compact, homogeneous layers with minimal intercellular spaces, resembling UCLs rather than a structured biofilm. None of the other non-adherent *S. cerevisiae* strains formed spatially structured biofilms; instead, they developed into compact UCL structures (similar to the UCLs formed by the BY4742 control) that varied only in thickness depending on the strain and medium, which likely correlates with the different growth ability of each strain in a given medium. Like the adherent strain YJM789, the non-adherent strains YJM145 and YJM451 showed limited growth in GM medium.

The results indicated that the ability of *S. cerevisiae* strains to adhere to plastic (Fig. 1) is generally associated with their ability to form a spatially structured biofilm (Fig. 4). None of the non-adherent strains produced a visually structured biofilm, whereas both adherent clinical strains (YJM320 and YJM789) and the BR-F strain did form spatially structured biofilms. However, the structure of these biofilms differed significantly between the clinical strains and BR-F, as did their adhesion characteristics. Neither clinical strain formed the typical wrinkled structures in GM medium (or on GMA) (Fig. 2), though hints of this structure were observed in the upper layers of YJM320 population, which grows better in GM than YJM789 (Fig. 4). It is possible that the lack of wrinkles is due to poorer growth of the clinical strains in respiratory GM; however, unlike BR-F, no wrinkling occurred in RPMI+glu, where both clinical strains grow well. In this medium, YJM789 produced a loosely organized structure with intercellular spaces, similar to the structures observed in both adherent clinical strains in YD. Thus, the medium composition not only differentially influences adhesion in different *S. cerevisiae* strains (Fig. 1A), but it also affects the internal organization of the resulting spatial biofilms (Fig. 4). This indicates differences in the regulatory pathways governing biofilm formation and cellular differentiation in response to environmental cues across different *S. cerevisiae* strains.

#### 2.4.2 Spatial structures of populations formed by different Candida species

The spatial structures formed by different *Candida* species differed significantly, with hyphae/pseudohyphae formation being a notable parameter influencing structural differences (Fig. 5). Three species, *C. parapsilosis*, *C. krusei*, and *C. albicans*, exhibited spatial structures composed of different cell types, including elongated hyphal/pseudohyphal structures, in at least one of the three tested media. The well adherent *C. krusei* formed spatially structured biofilms in all three media, with notable differences in internal biofilm organization between respiratory GM and the glucose YD and RPMI+glu media. In GM, hyphal/pseudohyphal structures were least prominent and appeared as large, radially organized cell clusters that were relatively loosely connected to the underlying cell layers. In contrast, in the glucose media, hyphae/pseudohyphae grew vertically from a basal layer composed of yeast-shaped cells.

Similarly, *C. parapsilosis* formed a structured biofilm in all three media (Fig. 5). This strain adhered relatively well in GM, with significantly reduced or negligible cell adhesion to plastic in YD and even lower in RPMI+glu (Fig. 1B). In GM, *C. parapsilosis* produced a distinct three-layer structure with hyphal/pseudohyphal cells concentrated in the middle (Fig. 5). In YD, the structures consisted of short hyphae or pseudohyphae emerging from basal cell layers, with occasional cell aggregates visible. Surprisingly, even in RPMI+glu, where *C. parapsilosis* exhibited almost no adhesion to plastic (Fig. 1B), it was still able to form a structured spatial population, although less prominent than in YD medium (Fig. 5). The complexity and robustness of the biofilm formed by *C. parapsilosis* was thus proportional to its adhesion efficiency in the tested media. On the other hand, this relatively weakly adherent *C. parapsilosis* strain consistently formed spatially structured biofilms in contrast to some *Candida* spp. strains that exhibited strong adhesion but did not form structured biofilms (see below).

*C. albicans* produced a biofilm structure only in RPMI+glu medium (Fig. 5), the only medium among those tested where it adhered well to the plastic surface (Fig. 1B). The *C. albicans* biofilm in RPMI+glu was substantial, consisting mainly of two layers of hyphae of different densities and lengths, and contained very few visible yeast-like cells (Fig. 5). Due to the compact nature of the lower hyphal layers, it was difficult to definitively determine whether the hyphae were directly attached to the plastic or whether a basal layer of yeast-shaped cells was present, as described in some *C. albicans* biofilm models [10,47]. Our current sample preparation method may have caused damage or detachment of hyphae, which are particularly long in *C. albicans* biofilms, from the plastic surface. To resolve this, further optimization of the method for this type of biofilm will be necessary. On GM and YD media, where *C. albicans* did not adhere, it formed compact UCLs composed solely of yeast-shaped cells. This layer was thin and discontinuous in GM, where *C. albicans* showed poor growth. The observed differences in cell filamentation between RPMI+glu and the other media are consistent with the previously described induction of *C. albicans* filamentation in RPMI [10,28,47].

For two species, *C. glabrata* and *C. tropicalis*, no hyphal/pseudohyphal formation was observed in any of the tested media. *C. glabrata* strain CBS138 and *C. tropicalis* adhered efficiently to plastic in all three media; however, only *C. glabrata* CBS138 developed a spatial structure with visually distinct cells in GM and, to some extent, in YD medium. The structure in GM resembled the pattern seen in *S. cerevisiae* strain YJM320, consisting of branched chains of cells with numerous intercellular spaces, growing from the dense basal cell layer. In YD, *C. glabrata* CBS138 formed a denser, more homogeneous structure, though there was still a visually apparent difference between cells in the basal and upper layers. No significant visual distinctions between cell layers were observed in highly adhesive strains *C. glabrata* CBS138 (in RPMI+glu) and *C. tropicalis* (in all media). These structures were similar to those of BY4742 and can therefore be classified as UCLs. The second, less adherent strain *C. glabrata* DSM11226 showed poor growth in GM and, despite moderate adhesion in RPMI+glu, did not form spatial structures with visibly distinct cells resembling thus the strain *C. glabrata* CBS138 in this medium. With our current approach, we cannot determine whether cells in some UCLs adhere to each other and/or whether morphologically uniform cells of UCLs exhibit spatial heterogeneity in other traits such as gene expression and metabolic activity.

Analyses of spatial structures formed by different *Candida* species demonstrated that the ability to adhere to plastic only correlates with the ability to form structured biofilms in certain cases, such as *C. albicans*. In contrast, some other *Candida* species with efficient adhesion to plastic, like *C. tropicalis*, did not form spatially organized biofilms according to our criteria. Conversely, the relatively low adherent *C. parapsilosis* developed prominent spatial structures similar to those of the highly adherent *C. krusei*.

## 3 Discussion

In this study, we compared the ability of yeast cells to adhere to surfaces and form spatially structured biofilms in selected *S. cerevisiae* strains, including clinical isolates, and in five *Candida* species (*C. albicans*, *C. glabrata*, *C. krusei, C. parapsilosis*, and *C. tropicalis*) in four different media. These media varied by only one parameter, either in their carbon source (glucose vs. glycerol in YD vs. GM) or in the presence or absence of yeast extract (YE) in the complex defined medium RPMI+glu.

### 3.1 Media composition and adhesion behavior of different yeast strains

Our results revealed prominent differences in adhesion efficiency between yeast species and strains and highlighted a complex relationship between medium composition and the physiological responses of adherent/non-adherent cells of different strains, beyond the simple distinction between respiratory (glycerol) and fermentative (glucose-based) media. Most notably, we observed a strong inhibitory effect of yeast extract on *C. albicans* adhesion. *C. albicans* DSM11225 adhered efficiently only when pre-grown in RPMI+glu without YE, while adhesion was nearly absent in all other media tested, including respiratory GM, glucose-based YD, and RPMI+glu+YE. This suggests the presence of an inhibitory component in YE that specifically affects *C. albicans* DSM11225 (and partially *C. glabrata* DSM11226) but does not interfere with adhesion in *S. cerevisiae* BR-F, clinical strains YJM789 and YJM320 or in other *Candida* species. Conversely, RPMI components significantly enhanced adhesion of *S. cerevisiae* BR-F and *C. krusei* DSM6128 in the presence of glucose. Relative adhesion of BR-F increased 3.8-fold and that of *C. krusei* 2-fold when comparing YD (glu+YE) and RPMI+glu+YE, media differing only by the RPMI components. In contrast, RPMI partially suppressed adhesion in the adhesive *S. cerevisiae* clinical strains YJM320 and YJM789 and this effect was counteracted by YE addition in RPMI+glu. These findings emphasize the high degree of phenotypic variability among *S. cerevisiae* strains under identical growth conditions likely reflecting differences in factors (and perhaps regulations) involved in cell adhesion. Similarly, YE improved adhesion in *C. tropicalis* and, to a lesser extent, *C. parapsilosis*, mimicking the response observed in *S. cerevisiae* clinical strains YJM789 and YJM320. These data underscore the need to further investigate the specific components in YE that inhibit *C. albicans* adhesion and those in RPMI that promote adhesion of *S. cerevisiae* BR-F and *C. krusei* in glucose-containing environments. Additionally, examining how different media influence planktonic cell physiology—particularly during cell transitions between growth phases—may shed light on effects of media components and mechanisms regulating cell physiology (e.g., dimorphic switching leading to yeast-to-hyphal transitions) and their impact on cell adhesion properties.

The marked variation in biofilm-forming ability observed between *S. cerevisiae* strains under identical growth conditions underscores the substantial phenotypic heterogeneity that exists between strains within a single species—an observation frequently reported in other yeasts, particularly *C. albicans* [5]. It is, however, challenging to directly compare the adhesion efficiencies of our yeast species with data from the literature, as the strains, media, conditions, and methods used for analyzing adhesion and biofilm formation vary significantly across studies. For example, our analyses showed strong adhesion of *C. krusei* and *C. tropicalis* in all media tested, including RPMI+glu, while *C. parapsilosis* showed low (or no) adhesion, except in GM medium (Fig. 1B). In contrast, Tan et al. (2016) [17] found good adhesion of *C. parapsilosis* across all media they used (RPMI with 0.2 % glucose, BHI [brain-heart infusion] medium, and glucose-rich YPD medium [YD with pepton]), while *C. krusei* adhered poorly (least in RPMI+0.2 % glu), and *C. tropicalis* showed the lowest adhesion. These discrepancies may reflect differences in strain origin - Tan et al. used strains isolated from voice prostheses of laryngectomized patients [17]. Other cell adhesion and biofilm studies, using the XTT (2,3-Bis(2-methoxy-4-nitro-5-sulfophenyl)-2H-tetrazolium-5-carboxanilide) reduction assays for adherent and biofilm cell quantification, showed, similar to our results, that different strains of *C. glabrata* respond differently to glucose concentrations [50]. The authors determined an optimal concentration of 0.2 % glucose for biofilm formation for two *C. glabrata* strains (including CBS138/ATCC2001), while a third *C. glabrata* strain showed minimal response to changes in glucose concentration. These experiments were performed in SD medium (synthetic defined minimal glucose medium), which lacks yeast extract. Our results show that YE can inhibit adhesion in *C. glabrata* DSM11226, while CBS138 strain adhered well in all media tested - including YD and RPMI+glu, both containing 2 % glucose (Fig. 1B). Interestingly, 2 % glucose inhibited adhesion of CBS138 in SD medium [50], suggesting that other media components may modulate glucose-dependent adhesion behavior. Further, adhesion and biofilm formation by *C. albicans* (strain ATCC10231) and *C. tropicalis* (strain DSM5991/ATCC13803), detected by measuring biofilm biomass grown from washed adherent cells and using crystal violet (CV) and MTT (tetrazolium salt3-[4,5-dimethylthiazol-2-yl]-2,5-diphenyltetrazolium bromide) assays, as well as scanning electron microscopy of the microtiter plate surface, revealed stronger cell adhesion (and biofilm formation) in RPMI 1640 medium (with 0.2 % glucose) but also significant adhesion and biofilm formation in YNB medium (with 100 mM glucose) [51].

Interestingly, *C. albicans* (strain DSM11225/ATCC90028 and three unrelated clinical strains) adhesion and biofilm formation in RPMI 1640 was strongly repressed by human serum in dose-dependent manner, and this effect was not caused by the proteinaceous components of the serum [52]. Human serum is a complex component like YE that inhibits *C. albicans* DSM11225 adhesion (Fig. 1B). Notably, other studies have shown that low concentrations of serum (3 %) can promote biofilm formation in *C. albicans* SC5314 in YNB medium [53] indicating strain-, medium-, and/or serum concentration-dependent effects.

### 3.2 Cell adhesion and spatial biofilm organization

Our results reveal a complex relationship between the ability of individual yeast strains to adhere to a solid surface—the first step of biofilm development—and their ability to form mature, spatially structured biofilms under defined growth conditions. The comparison of *S. cerevisiae* strains revealed that those strains capable of efficient adhesion to plastic (the natural strain BR-F and some clinical strains) were also able to form spatially structured biofilms in a given medium. However, the biofilm architecture varied depending on the specific strain, and different media had different effects on the adhesion and biofilm formation capabilities of these strains. Contrary to findings that question the ability of *S. cerevisiae* to form biofilms [10] and consistent with Van Nguyen et al. [15], new results support that the BR-F strain, as well as some other *S. cerevisiae* strains, are capable of biofilm formation. Importantly, this conclusion is based not only on the ability of *S. cerevisiae* to adhere to surfaces, as demonstrated in various studies [[11], [12], [13], [14]], but also on its capacity to form a 3D biofilm with a pronounced internal structure (Fig. 1A and Fig. 4). *S. cerevisiae* can therefore serve as an excellent model for biofilm research, especially given the availability of numerous tools for manipulating this yeast. A key challenge for future research will be to identify the genetic determinants that drive strain-specific differences in biofilm architecture under particular environmental conditions (media).

In contrast, the relationship between cell adhesion to plastic and the structuring of the mature biofilm was less uniform among the different *Candida* species. In *C. albicans* and *C. krusei*, as in *S. cerevisiae*, efficient adhesion to plastic was associated with the formation of spatially structured biofilms. However, this relationship does not apply to *C. tropicalis* and *C. glabrata* CBS138, which adhere well to plastic in all media but either do not form spatially structured biofilms in any medium (*C. tropicalis*) or only in certain media (*C. glabrata*). This relationship also does not apply to *C. parapsilosis*, which was able to form a well-structured spatial biofilm, although it exhibited little to no adhesion to plastic in RPMI+glu. As defined above, we consider a spatially structured biofilm as a three-dimensional structure consisting of morphologically distinct cells – such as yeast cells and differently elongated hyphae or pseudohyphae – and in some cases also exhibiting macroscopic wrinkling. Although some efficiently adherent strains (e.g., *C. tropicalis*) did not produce such complex structures, instead forming morphologically homogeneous cell layers (UCLs) similar to non-adherent strains (e.g., *S. cerevisiae* BY4742), we cannot exclude that intercellular adhesion and metabolic heterogeneity may still occur at a physiological level, without being reflected in cell morphology. Identifying such functionally distinct but morphologically indistinguishable subpopulations within biofilms remains a significant challenge for future research. Addressing this will require a deeper understanding of the metabolic processes active during biofilm development and, building on that knowledge, the implementation of fluorescently labeled protein markers or specific dyes to visualize spatially regulated cell subpopulations within the biofilm structure. For instance, studies in *S. cerevisiae* have shown that biofilms may contain small, specialized subpopulations—such as cells expressing multidrug resistance (MDR) transporters—that localize near the biofilm surface and contribute to the biofilm protection [44].

Currently, information regarding the internal structure of biofilms formed by various yeast species and strains is limited. The relatively thick biofilm structure and the presence of a cell wall in yeast cells (which scatters light or laser beam during confocal microscopy) complicate the analysis of internal structure when viewed from the top. For example, studies using scanning electron microscopy often visualize only cells adherent to the surface [51,54] rather than the internal structure of the mature biofilm. In this study, we employed an approach of cross-sections to visualize native biofilm structures from the side, enabling insight into the vertical organization of cell layers throughout the full biofilm depth. However, our method originally developed for *S. cerevisiae* biofilms [15], may not fully preserve long, fragile hyphal structures, such as those formed by *C. albicans* in RPMI+glu. Previous images of *C. albicans* biofilm vertical structure, obtained after biofilm fixation and staining with Calcofluor White and imaging using two-photon confocal microscopy (which allows deeper penetration into the biofilm using z-stacks from 500 scans) and side view of projection images [47], showed a biofilm structure in RPMI similar to what we observed in our analysis. However, the authors reported the presence of a basal layer of yeast-shaped cells from which long hyphae emerge, a feature we have not yet detected. This discrepancy could be due to the high density of hyphae, which makes the preparation of vertical sections difficult, or to differences in the media used for cultivation - our study used RPMI with 2 % glucose, while the earlier study used RPMI with 0.2 % glucose [47].

In the most comprehensive study to date, Mancera et al. analyzed the formation and structure of biofilms in several yeast species, including *C. albicans*, *C. tropicalis*, *C. parapsilosis*, *S. cerevisiae*, and *C. glabrata*, in RPMI and Spider media with various glucose concentrations or supplemented with galactose [10]. The authors identified Spider+1 % glucose as the best adhesion medium and RPMI+2 % glucose as the second best. To visualize the side views of biofilm, they used z-stacks from confocal scanning microscopy. Consistent with our findings, *C. albicans* formed long hyphae in RPMI+glu. However, different results were reported for *C. tropicalis*, *C. parapsilosis*, and *S. cerevisiae*. *C. tropicalis* formed long hyphae in their RPMI+2 % glucose experiments [10], whereas we observed no filamentation or structured biofilm by this species. Mancera et al. also found weaker biofilm structuring in *C. parapsilosis* in RPMI+glu compared to our observations and reported that *S. cerevisiae* did not form biofilms [10]. These differences may reflect strain-specific behavior. The *C. albicans*, *C. tropicalis*, and *S. cerevisiae* strains used in our study differed from those in Mancera et al. [10], whereas *C. parapsilosis* strain (DSM5784/CLIB214/ATCC22019) shared a common genetic background but originated from different sources. Such variation supports the idea that biofilm-forming capabilities - and associated structural phenotypes - can differ even among genetically similar strains. In the case of *C. parapsilosis* (DSM5784/CLIB214/ATCC22019), observed differences in biofilm formation in the same medium (RPMI+glu) suggest a rapid divergence of biofilm-forming strategies, possibly driven by the accumulation of spontaneous mutations. Strain-specific variation in different traits, even among genetically related isolates from different environments, has been previously documented [5].

Other studies on biofilm formation in various media typically do not provide sufficient information on the internal biofilm structure. For example, Arevalo-Jaimes et al. (2023) [26] used four different complex media (RPMI, YPD, BHI, and TSB [tryptic soy broth]) with different glucose concentrations to analyze biofilm formation in *C. parapsilosis*. They detected biofilms in all media, which differed in various parameters (e.g., filamentation, metabolic activity, etc.), but the confocal microscopy z-stacks used in their study did not allow to distinguish the spatial arrangement or morphologically distinct cell types within the biofilms.

The different yeast behaviors in terms of cell adhesion and biofilm formation may be influenced by different mechanisms regulating cell metabolism in response to nutrient availability and the genetic background of the individual strains. Additionally, these behaviors can be modulated by interactions with other microorganisms in multispecies biofilms, where mutual influences may significantly alter structural and functional biofilm characteristics [55]. Such interactions, along with the overall composition of the colonized niche, can profoundly affect adhesion dynamics and biofilm development. The remarkable variability in cell adhesion and biofilm architecture - both within and between yeast species - underscores the adaptability of yeasts to diverse and changing environments. This phenotypic plasticity enables yeasts to occupy a wide range of ecological niches and to dynamically modify their behavior based on local conditions.

Although experimental biofilms are grown under simplified and defined laboratory conditions that differ from the complex and dynamic environment within a host, these controlled systems allow the precise identification of molecular mechanisms and environmental triggers. Some of these factors may also be relevant under natural conditions. For instance, the availability of glucose is a well-established factor influencing biofilm formation [26,50,56], and the increased risk of invasive fungal infections in diabetic patients [[57], [58], [59]] underscores the clinical relevance of such metabolic cues. The differential effects of YE and RPMI components between *S. cerevisiae* and *C. albicans* document that beyond primary carbon sources, specific medium components can trigger distinct cellular responses in different yeasts. To clarify these influences, further detailed studies are necessary to identify the key active components within the YE and RPMI and investigate their impact on cellular physiology throughout the biofilm development process. This knowledge could then make it possible to identify the responsible genetic determinants and regulations.

Identifying critical metabolic and regulatory pathways that govern adhesion and biofilm maturation in controlled experimental conditions could facilitate the design of more effective biofilm control strategies - for instance for medical applications, such as decontamination of medical devices. Importantly, such research could also contribute to a better understanding of biofilm formation in natural environments, including host-associated niches.

## 4 Material and methods

### 4.1 Yeast strains and growth media

The yeast strains used in this study are listed in Table 1. The media used included respiratory GM medium (3 % glycerol, 1 % yeast extract), and glucose-based media YD (2 % glucose, 1 % yeast extract), RPMI+glu (RPMI 1640 with glutamine and phenol red, with bicarbonate [LM-R1640, BIOSERA, France]) supplemented with D-glucose to a final concentration of 2 %, pH adjusted to 5.6 with a few drops of 1 M HCl [28], RPMI+glu+supplements (RPMI+glu with 50 mg/l of histidine, leucine, and lysine, and 20 mg/l of uracil) for cultivation of *S. cerevisiae* BY4742 auxotroph, and RPMI+glu+YE (RPMI+glu supplemented with 1 % yeast extract). For colony morphology experiments, solid agar media were used: GMA (GM with 2 % agar), YDA (YD with 2 % agar) and RPMI+gluA (RPMI+glu with 2 % agar).

### 4.2 Cell cultivation for adhesion assay

The cells were inoculated with an initial density of A_600_ = 0.05 or 0.01 (Helios Gamma spectrophotometer, Unicam) depending on the strain. After incubation at 28 °C with shaking at 150 rpm, the cells were harvested and washed with sterile water. For strains grown in glucose-containing media (YD, RPMI+glu, and RPMI+glu+YE), the glucose level was monitored prior to harvesting for the adhesion assay. The incubation time was adjusted so that glucose was still present in the medium at the time of cell harvest, which was checked using the GlukoPhan test (PLIVA-Lachema Diagnostika s.r.o. Brno). In this way, we were always able to ensure that the cell culture used for adhesion was in a phase in which it used glucose as a carbon source and had not yet entered the post-diauxic phase, in which the glucose is depleted and the cells switch metabolically to other sources. For most cultivations, a 14-h incubation time was used, which was only shortened to 4–6.5 h in the case of the richer RPMI+glu+YE medium, as the strains grew faster there than in the other media. The harvested cells were then resuspended in distilled water to A_600_ = 1, and 150 μl of the cell suspension was transferred into the wells of a 96-well microtiter plate, with four independent cell replicates per strain and medium. The plates were incubated at 28 °C with shaking for 3 h before adhesion was measured.

### 4.3 Adhesion assays and data processing

Cell adhesion to polystyrene wells was quantified using a modified adhesion assay based on methods from Refs. [13,60]. After incubation, the liquid portion of the cell suspension was removed from the microplates, and the wells were washed three times with distilled water. Each well was then incubated with 150 μl of 1 % crystal violet dye at room temperature with gentle shaking for 15 min. The dye was then removed, and the wells were washed three times with distilled water. The plates were air-dried for 30 min, and the dye retained by the adherent cells was eluted with 150 μl of a 2:1 mixture of 96 % ethanol and distilled water for 1 min. The absorbance (A_592_) of the eluted dye was measured using an Epoch Microplate Spectrophotometer BioTek (Agilent, US) after 100 μl of the eluted solution was transferred to a new microtiter plate. The absorbance values corresponded to the quantity of adherent cells and were used as a measure of relative adhesion efficiency. Control wells, where the complete procedure was conducted in the absence of cells, were used to account for background staining, and these values were subtracted from the measured values.

Relative adhesion was calculated relative to the adhesion of the *S. cerevisiae* BR-F strain in GM medium, which was set at 100 %. Standard deviation was calculated from four replicates, and the statistical significance of differences was determined using a one-way ANOVA test with Tukey post test for multiple comparisons and GraphPad Prism 6 software. P values of 0.05 or less were considered statistically significant: ∗, P < 0.05; ∗∗, P < 0.01 and ∗∗∗, P < 0.001.

### 4.4 Cell cultivation for biofilm imaging and microscopy

Strains were inoculated at the same optical density as for the adhesion assay. After 14 h of growth, the cell suspension was centrifuged, and the cells were resuspended in fresh medium at an A_600_ = 0.36. Subsequently, 250 μl of the cell suspension was inoculated into each well of a microtiter plate, with four to twelve independent cell replicates per strain and medium. The plates were incubated for 24 h at 28 °C.

### 4.5 Biofilm imaging and cell microscopy

Images of structures in the microtiter plate wells (top view) were captured using a Basler acA2440-75ucMED camera (Basler MED ace 5.1 MP, 75 color) equipped with a Navitar objective. The images were processed using NIS Elements software (Laboratory Imaging, s.r.o., Prague, CZ).

For microscopy of the internal vertical cell arrangement, SLI structures (biofilms and UCLs) were embedded in 4 % agarose to stabilize their structure and sectioned using a Leica VT1200S vibrating microtome according to the method in Van Nguyen et al. (2020) [15]. Sections of biofilms and UCLs (section thickness typically 20 μm, for *C. albicans* 40 μm) were observed with a Carl Zeiss Axio Observer.Z1 inverted fluorescence microscope equipped with an Axiocam 506 camera and a *C*-Apochromat 10×/0.45 W objective using the ZEN 2012 (blue edition) software. In each of the two independent biological experiments, cross-sections of at least two independent SLI structures were analyzed. Fig. 4, Fig. 5 show a representative part of the entire biofilm/UCL cross-section (over the entire diameter of the well in the plate), which contained all the structures observed in the respective biofilm/UCL, including their relative representation. It is important to note that this method allows the analysis of all structures (biofilms and UCLs), regardless of whether the cells within the structures adhere to each other and/or whether the cells adhere to the bottom of the plastic chamber.

## CRediT authorship contribution statement

**Vichi Sicha Irianto:** Methodology, Investigation, Formal analysis, Data curation. **Vítězslav Plocek:** Methodology, Investigation, Formal analysis, Data curation. **Rashim Bharti:** Investigation, Data curation. **Jana Maršíková:** Investigation, Data curation. **Libuše Váchová:** Writing – review & editing, Supervision, Formal analysis, Conceptualization. **Zdena Palková:** Writing – review & editing, Writing – original draft, Supervision, Funding acquisition, Formal analysis, Conceptualization.

## Declaration of competing interest

The authors declare that they have no known competing financial interests or personal relationships that could have appeared to influence the work reported in this paper.

## Data Availability

This study does not generate data deposited in external repositories. The data that support the findings of this study are available from the corresponding author upon reasonable request.
